# Effects of Aging on Formalin-Induced Pain Behavior and Analgesic Activity of Gabapentin in C57BL/6 Mice

**DOI:** 10.3389/fphar.2020.00663

**Published:** 2020-05-08

**Authors:** Damiana Scuteri, Laura Berliocchi, Laura Rombolà, Luigi Antonio Morrone, Paolo Tonin, Giacinto Bagetta, Maria Tiziana Corasaniti

**Affiliations:** ^1^Preclinical and Translational Pharmacology, Department of Pharmacy, Health Science and Nutrition, University of Calabria, Cosenza, Italy; ^2^Department of Health Sciences, University “Magna Graecia” of Catanzaro, Catanzaro, Italy; ^3^Regional Center for Serious Brain Injuries, S. Anna Institute, Crotone, Italy

**Keywords:** aging, behavioral and psychological symptoms of dementia, dementia, pain, gabapentin, formalin test

## Abstract

Improved living conditions have induced an increase of lifespan often accompanied by comorbidities, responsible for pain, and by cognitive impairment and dementia, impairing communication capabilities. In most cases, the elderly do not receive pain relief because of underdiagnosis and of aging-induced changes of systems affecting nociceptive response. Unrelieved pain is involved in the development of behavioral symptoms, as agitation, representing a difficult challenge in this fragile population. Aged C57BL/6 mice and amyloid precursor protein (APP) mice display behavioral disturbances that mimic behavioral and psychological symptoms of dementia (BPSD). Therefore, this original study focuses on the influence of aging on nociception to provide insight into the occurrence of BPSD. We have investigated how aging can affect nociception after formalin administration and gabapentin effect in C57BL/6 mice, since it represents one of the treatments of choice for chronic neuropathic pain. Based on our results, changes of nociceptive behavior in response to an algogen stimulus occur during aging. Formalin-induced behavioral pattern in older C57BL/6 mice presents a temporal shift and an increase in the peak amplitudes. Our data show that the effectiveness of gabapentin is influenced by the age of the animal; though preliminary, the latter provide evidence upon which formalin test induced long-lasting mechanical allodynia might be a reliable as rapid and viable persistent pain model. The disclosed differences in effectiveness of gabapentin according to age can form the rational basis to deepen the study of pain treatment in the elderly.

## Introduction

During the last 150 years the improvements in medicine and in standards of living induced an increase of three months per year in life expectancy at birth (Scully, 2012). Unfortunately, longevity and aging predispose to chronic and progressive neurodegenerative conditions. In particular, 50 million people worldwide suffer from dementia and this number will likely triple by 2050 (Patterson, 2018). The most affected segment of population is represented by adults older than 65 years, often presenting comorbidities responsible for pain states, (see (Scuteri et al., 2018b)) e.g. arthritis, herpes zoster, diabetes accompanied by neuropathy and retinopathy (see (Scuteri et al., 2019b)) and migraine, disabling (see (Scuteri et al., 2019a)), and difficult to treat (see (Scuteri et al., 2020)). Up to 80% of patients resident in nursing homes often shows moderate to severe pain (see (Sandvik et al., 2014)). Unrelieved pain might be a contributory factor for the development of agitation (Husebo et al., 2011; Sampson et al., 2015). Poor communication skill of patients affected by dementia is an important determinant of underdetected pain (Kovach et al., 2005; Scuteri et al., 2017a). The latter problem makes the assessment of pain very difficult in this fragile population, thus receiving less pain medication than cognitively intact elderly (Horgas and Tsai, 1998; Achterberg et al., 2013). Indeed, the treatment of pain with a stepwise protocol is demonstrated to reduce agitation at the Cohen-Mansfield agitation inventory of the 17% (Husebo et al., 2011). Intense agitation and anxiety are the main features of the behavioral and psychological symptoms of dementia (BPSD) known under the definition of sundowning syndrome, frequent challenge in institutionalized demented and normal elderly (Evans, 1987; Bachman and Rabins, 2006; Bedrosian et al., 2011). Mice subjected to spared nerve injury develop depressive-like behavior and cognitive dysfunction with significant enhancement in β-amyloid 1–40 serum peptide levels (D’Aniello et al., 2017). Incidentally, aged C57BL/6 mice and amyloid precursor protein (APP) mice both present an anxiety pattern of behavior that mimics this BPSD (Bedrosian et al., 2011). Aging is associated with changes of the systems involved in nociception (Hamm and Knisely, 1985; Hamm and Knisely, 1986), as well as dementia (Parvizi et al., 2000; Zarow et al., 2003). How aging impacts on pain processing and on painkillers effectiveness has not been well understood yet. Indeed, the lack of homogeneous results yielded so far may stem from differences in strain, age, and test for sensitivity assessment (Yezierski, 2012). Aged Lou/c/jall rats present increased mechanical sensitivity to Von Frey’s test and paw pressure test (Jourdan et al., 2000; Jourdan et al., 2002). Older rats show more sensitivity to cold and morphine is less effective in producing antinociception during thermal, hot, stimulation. In particular, aged Fischer 344 × Brown Norway F1 rats placed in an apparatus with temperature-controlled floor plates spend more time in the floor at the neutral temperature of 30°C as compared to hot (45°C) and cold (15°C) temperatures. Aversion is greater to cold than to heat, but it is increased by exposure to extreme hot and cold temperatures with significant influence of age (Morgan et al., 2012). Among others, we have used the formalin test (Dubuisson and Dennis, 1977) because of favorable features. In fact, following a period of inflammation, it induces longer lasting hyperalgesia. In particular, peripheral inflammation and nocifensive behavior ensue immediately after the administration of formalin, whilst hyperalgesic response occurs after 2 h and builds up from the first to the third day lasting 3 to 4 weeks (Fu et al., 2001; Guida et al., 2012). Formalin-induced licking/biting behavior is characterized by an early nociceptive phase and a late phase in which central sensitization occurs (see (Scuteri et al., 2018a)). Interestingly, the α2δ-1 calcium channel subunit, important for channel assembly, is overexpressed during central sensitization and allodynia in a number of specific pain models (Luo et al., 2002). The α2δ-1 ligands, known as gabapentinoid drugs, represent a largely validated approach for chronic neuropathic pain treatment (Scuteri et al., 2017b). Therefore, here we aim at characterizing the impact of aging on formalin evoked nociception and gabapentin efficacy in C57BL/6 mice.

## Materials and Methods

### Animals

Male C57BL/6 mice (Charles River, Italy) of 2, 6, 13, and 20 months of age at the beginning of the experiment have been used. Mice have been housed in groups of 4 per cage on a 12 h:12 h light dark cycle at constant room temperature of 22 ± 1°C and in conditions of relative humidity of the 65% and provided with food and water *ad libitum*.

Ethical review and approval was not required for the animal study because the experimental protocol is in accordance to the European Community Council Directive of 24 November 1986 (86/609/EEC) and L.D. 4 March 2014 No. 26 has been followed to minimize the number of animals used still generating reliable results. Since this project has been approved when the D.M. 116 was still in validity, no other approval was required.

The severity of the formalin test procedure is slight (for pain intensity and duration) according to the annex VII of the L.D. 26 quoted in the experimental procedure section. More importantly, our work has used this test to replace more severe surgical procedures to mimic persistent pain. Similarly, the very low number of animals used in this behavioral study cannot be reduced further. Indeed, the experimental design, considering group sizes and statistical power analysis, balances the need for reliable results while keeping the number of animals as low as possible. Accordingly, we meet with the scope of the 3R approach to refine, reduce, and, at least in part, replace. Based on statistical power calculation and according to similar studies in literature, n=5 animals per group subjected to gabapentin treatment is sufficient to obtain 30% reduction of formalin-induced mechanical allodynia. Therefore, this n has been chosen to use the minimum number of animals still generating reliable results.

### Experimental Pain Model

The experimental pain model is the formalin test (Dubuisson and Dennis, 1977). Mice are allowed to acclimatize in a plexiglas box (30 x 30 x 35 cm^3^) for up to 60 min maintaining room temperature and humidity stable. Twenty µl of a 5% solution of fresh formalin obtained from a solution of saturated formaldehyde at 36.5–38% (Sigma F8775) were administered subcutaneously (s.c.) into the left hindpaw of the mouse. The licking/biting/flinching behavior is monitored for 90 min at intervals of 5 min.

### Behavioral Test

The Von Frey’s test (Chaplan et al., 1994) is performed to assess mechanical allodynia. For acclimation, mice are placed inside perspex chambers (75 mm x 90 mm) on a wire mesh floor for up to 60 min. This test uses calibrated filaments, the Von Frey’s hairs (Ugo Basile, Comerio, Italy), through the up-down method (Dixon, 1980; Chaplan et al., 1994) that allows to determine the value corresponding to the 50% of the withdrawal threshold. The method for calculation is based on “k value” and “log final hair”, considering the stiffness of the Von Frey’s hair. In particular, the 50% response threshold is interpolated through the formula “50% g threshold = (10 ^[Xf+kδ]^)/10.000” where: Xf = value (in log units) of the final Von Frey’s hair used; k = tabular value for the pattern of positive/negative responses; δ = mean difference between stimuli expressed in log units. Von Frey’s hairs have logarithmically incremental stiffness (0.41, 0.70, 1.20, 2.00, 3.63, 5.50, 8.50, and 15.10 g) (Chaplan et al., 1994). During the behavioral tests the room temperature and humidity are maintained constant. The timeline of the behavioral tests is as follows:

**Figure:**
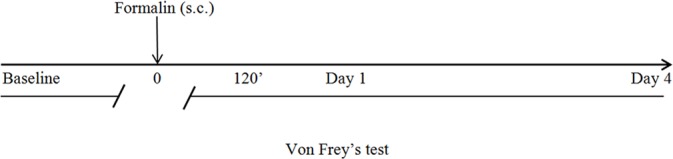


### Drug Treatment

Gabapentin/vehicle is administered intraperitoneally (i.p.) 15 min before formalin injection. Gabapentin is used in two different doses: 10 mg/kg or 100 mg/kg. Based on the existing literature, the dose of 100 mg/kg has been selected and compared with a second 10-fold lower dose (10 mg/kg) and a vehicle. Gabapentin is dissolved in depurated water (vehicle) according to its solubility (10 mg/ml). The Von Frey’s test is performed before the administration of formalin in order to get the baseline threshold. To assess the effect of gabapentin on formalin-induced mechanical allodynia in mice of different ages, the Von Frey’s test is performed 2 h after the administration of formalin, on the following day and on the 4^th^ day after formalin test. In view of the reported circadian oscillation of α2δ-1 subunit expression and variability of response to gabapentin, our experiments started at 9:30 a.m. in all instances (Kusunose et al., 2010).

### Statistical Analysis

Data are expressed as mean ± SEM and assessed statistically for differences by two way analysis of variance (ANOVA) followed by Bonferroni’s multiple comparisons test (GraphPad Prism). p values < 0.05 are considered statistically significant.

## Results

### Effect of Aging on Formalin-Induced Licking/Biting/Flinching Behavior

**Figure 1:**
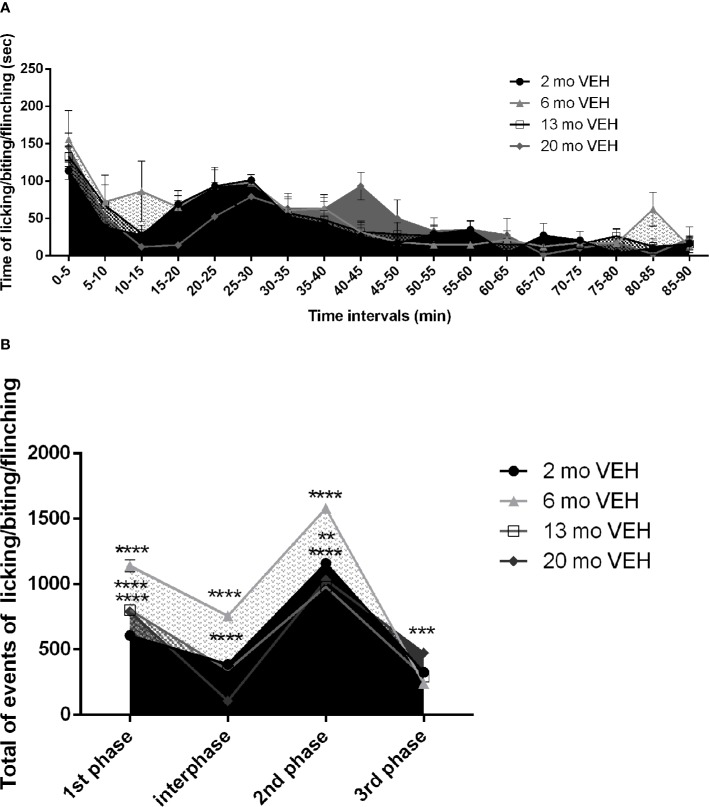
Effect of aging on formalin-induced licking/biting/flinching behavior. The first phase occurs in the first 5 min in all the age groups, while the second phase is shifted and prolonged (30–45 min) in aged 20 months mice (**A**. Two-way ANOVA F (17, 216) = 15,69; p < 0,0001**** for time points factor). The amplitude of the peak in the first phase increases in 13 and 20 month-old mice, but it reaches the highest level in mice of 6 months (**B**. Two-way ANOVA F (9, 52) = 60,02; p < 0,0001****: 2 vs 6 months p< 0,0001****; 2 vs 13 months p< 0,0001****; 2 vs 20 months p< 0,0001****). Also in the interphase (**B**. Two-way ANOVA F (9, 52) = 60,02; p < 0,0001****: 2 vs 6 months p< 0,0001****; 2 vs 20 months p< 0,0001****) and in the second phase (**B**. Two-way ANOVA F (9, 52) = 60,02; p < 0,0001****: 2 vs 6 months p< 0,0001****; 2 vs 13 months p< 0,0001****; 2 vs 20 months p< 0,01**) the highest amplitude occurs in 6 month-old mice. The 20 month-old mice, they develop a higher third phase (**B**. Two-way ANOVA F (9, 52) = 60,02; p < 0,0001****: first phase 2 vs 20 months p< 0,001***). Data are expressed as mean ± SEM of the nociceptive reaction. p values <0.05 were considered statistically significant.

The formalin test provides an initially inflammatory stimulus turning into a persistent pain trigger in the long-term and formalin-induced nocifensive behavior undergoes modifications both in the intensity and in the duration with increasing age of the animal. While the 2 month-old mice show the behavioral pattern typically induced by formalin (Dubuisson and Dennis, 1977), the curves of older mice show a temporal shift (**Figure 1A**) with an increase in the peak amplitudes (**Figure 1B**). Under these experimental conditions, due to the detected different duration of the behavioral pattern in aged mice, the latter has been monitored for 90 min, instead of the classic 60 min. While the first phase occurs in the first 5 min in all the age groups, the second phase, presented by 2 month-old mice from 25 to 30 min since formalin injection, is shifted and prolonged (30–45 min) in 20 months mice (**Figure 1A**. Two-way ANOVA F (17, 216) = 15,69; p < 0,0001**** for factor time). This likely indicates an increased latency to recovery. Moreover, the amplitude of the peak in the first phase increases in 13 and 20 month-old mice, but it reaches the highest level in mice of 6 months (**Figure 1B**. Two-way ANOVA F (9, 52) = 60,02; p < 0,0001****: 2 vs 6 months p< 0,0001****; 2 vs 13 months p< 0,0001****; 2 vs 20 months p< 0,0001****). Also in the interphase (**Figure 1B**. Two-way ANOVA F (9, 52) = 60,02; p < 0,0001****: 2 vs 6 months p< 0,0001****; 2 vs 20 months p< 0,0001****) and in the second phase (**Figure 1B**. Two-way ANOVA F (9, 52) = 60,02; p < 0,0001****: 2 vs 6 months p< 0,0001****; 2 vs 13 months p< 0,0001****; 2 vs 20 months p< 0,01**) the highest amplitude occurs in 6 month-old mice. It is conceivable that the prolonged response of 20 month-old mice may yield the observed higher third phase (**Figure 1B**. Two-way ANOVA F (9, 52) = 60,02; p < 0,0001****: first phase 2 vs 20 months p< 0,001***).

### Influence of Aging on Gabapentin Effects in Formalin Test

**Figure 2:**
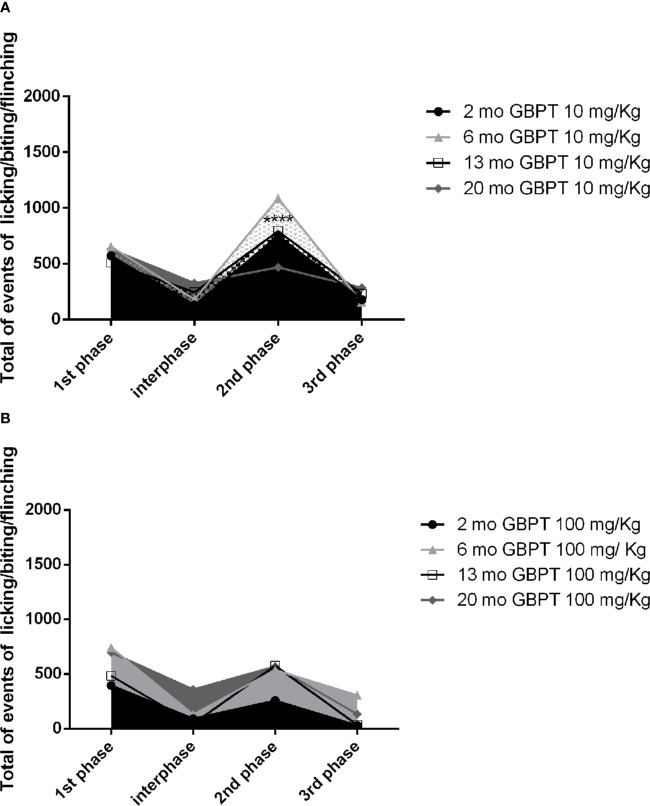
Influence of aging on gabapentin effects in formalin test. In the second phase the low dose of 10 mg/kg of gabapentin is more active in 20 than in 2 month-old mice (**A**. Two-way ANOVA F (9, 56) = 88,16; p < 0,0001****: 2 vs 20 months p< 0,0001****). On the contrary, the higher dose of 100 mg/kg is more effective in the 2 month-old mice rather than in all the groups of older mice (**B**. Two-way ANOVA F (9, 64) = 56,54; p < 0,0001****). Data are expressed as mean ± SEM of the nociceptive reaction. p values <0.05 were considered statistically significant.

The effect of gabapentin on the nociceptive response induced by formalin is influenced by the age of the mice. A low dose, poorly effective or ineffective (Dixit et al., 1999) in young adult rodents, shows increased efficacy in aged mice. In fact, in the second phase the low dose of 10 mg/kg is more active in 20 month-old than in 2 month-old mice (**Figure 2A**. Two-way ANOVA F (9, 56) = 88,16; p < 0,0001****: 2 vs 20 months p< 0,0001****). At variance with the latter, the dose of 100 mg/kg results more effective in 2 month-old (**Figure 2B**. Two-way ANOVA F (9, 64) = 56,54; p < 0,0001****) mice but not in all of the other age groups of mice.

### Influence of Aging on Gabapentin Effects in Formalin-Induced Mechanical Allodynia

**Figure 3:**
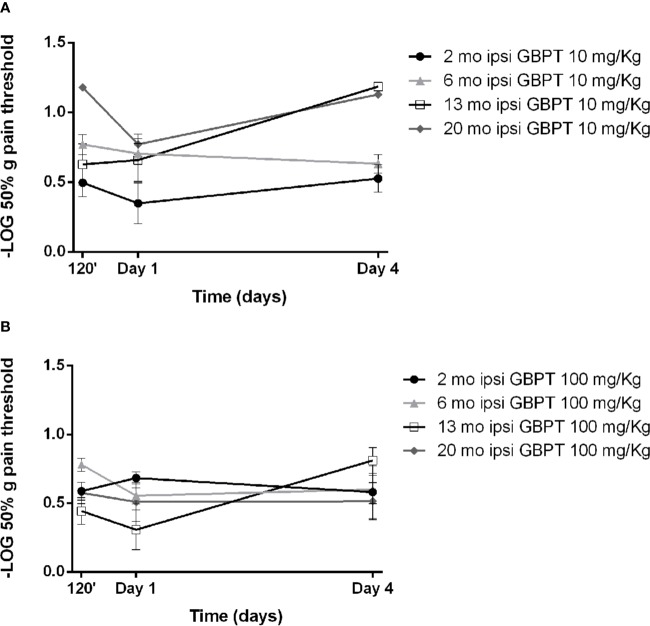
Influence of aging on gabapentin effects in formalin-induced mechanical allodynia. The lower dose of gabapentin is not active (**A**. Two-way ANOVA F (9, 56) = 2,925; p = 0,0065 **) and the higher dose is more effective in aged mice (**B**. Two-way ANOVA F (9, 64) = 1,930; p = 0,0633). Data are expressed as −LOG of the mean ± SEM of the 50% pain threshold. p values <0.05 were considered statistically significant.

The effects of systemic pretreatment with gabapentin have been studied on tactile allodynia induced by formalin. A pretreatment with 10 mg/kg of gabapentin is ineffective in all age groups (**Figure 3A**. Two-way ANOVA F (9, 56) = 2,925; p = 0,0065 **). The higher dose (100 mg/kg) appears to be more effective in aged mice, though this effect does not reach statistical significance (**Figure 3B**. Two-way ANOVA F (9, 64) = 1,930; p = 0,0633).

## Discussion

Aging and dementia are often accompanied by comorbidities responsible for underdiagnosed chronic pain which is often associated to the development of BPSD, in particular agitation (Husebo et al., 2011). Memantine, through an uncompetitive/fast-off rate action mainly on extrasynaptic N-methyl-D-aspartate (NMDA) receptors (see (Scuteri et al., 2017c)), delays progression of disease but does not prevent agitation from occurring. Aged mice show behavioral disturbances comparable to those displayed by APP mice (Bedrosian et al., 2011; Bedrosian and Nelson, 2013). Therefore, the purpose of this preclinical study is to deepen the knowledge concerned with the effects of aging on a persistent pain condition alluding to a clinically relevant state and on the efficacy of gabapentin. Aged C57BL/6 mice show a different trend of the classical formalin-induced behavioral pattern with a shift in time and amplitude, thus supporting the occurrence of modifications in the mechanisms of central sensitization. These results are in agreement with the evidence that aged animals develop heightened hypersensitivity in several experimental pain models (Crisp et al., 2003; Bishay et al., 2013) and that formalin test behavioral pattern is influenced by age (Gagliese and Melzack, 1999). Also under different experimental conditions (10 μl of 4% formalin solution (Kolber et al., 2010)), C57BL/6 mice display enhanced nociceptive behavior and increased variability among different ages in the second phase and mechanical allodynia (Sadler et al., 2017). Aged C57BL/6J mice present a mitochondrial impairment (Kadoguchi et al., 2020) and a decrease in oxidative phosphorylation as well as alteration in apoptosis regulation (see (Azzu and Valencak, 2017)). Caspase activation and apoptosis is involved in the neuropathogenesis of Alzheimer’s disease (Su et al., 1994; Su et al., 1997; Barnes et al., 1998; Mattson, 2002; Shao et al., 2014). Interestingly, aged C57BL/6 mice also display neurobehavioral changes (Dean et al., 1981; Traschutz et al., 2018). In the clinic, aging causes a decrease of pain tolerance threshold (Lautenbacher, 2012; Paladini et al., 2015) and an impairment of descending modulatory pathways (Washington et al., 2000; Riley et al., 2010; Paladini et al., 2015). Neuropathological alterations can impact nociception at a great extent and according to the type of dementia (Scherder et al., 2003). Central sensitization and plastic modifications occurring at level of the dorsal horn (Tjolsen et al., 1992), likely implicated in the formalin-induced second phase and late long-term mechanical allodynia (Fu et al., 2001; Guida et al., 2012), could undergo modifications with the increase of age of the animal. Formalin test, using mainly 5% formalin induces concentration-dependent hypersensitivity resembling neuropathic pain induced by spinal nerve injury enhancing α2δ−1 subunit protein levels in dorsal root ganglia (Salinas-Abarca et al., 2017). Moreover, formalin-induced allodynia is reversed by gabapentin as allodynia induced by spinal nerve ligation (Salinas-Abarca et al., 2017). In our experimental setting, mechanical allodynia induced by formalin test shows different features according to the age of the animal, with an apparent more difficult recovery from injury in 13 and 20 month-old mice; this is likely linked to the observed different basal threshold of these mice according to their age. In agreement with previous data (Fu et al., 2001; Guida et al., 2012), here we have reported that the hindpaw contralateral to formalin injection develops mechanical allodynia on the day 4^th^, supporting the deduction that central sensitization mechanisms are implicated. Quite importantly, a reportedly poorly active or inactive (Dixit et al., 1999) dose of gabapentin here shows stronger efficacy in formalin-induced nociceptive, but not in mechanical allodynia, response in aged mice. The latter pharmacological responses may be suggestive of the expression of a behavioral component depending on α2δ-1 subunit expression, conceivably subjected to quantitative variability according to age and phase of the formalin test. Additional studies are needed to strengthen our preliminary evidence and to dissect the molecular basis of the behavioral and pharmacological responses described here. It is conceivable that performing formalin test in transgenic 3xTg-AD mice, bearing an age-dependent cognitive and behavioral profile (Baeta-Corral et al., 2018), might help disclosing the effect of pain on behaviors recapitulating BPSD.

## Data Availability Statement

All datasets generated and analyzed for this study are included in the manuscript.

## Author Contributions

LB, GB, PT, LM, and MC conceived the study. DS participated in the conceptualization of the study, carried out the experiments, analyzed the results, and wrote the manuscript. LR participated to the analysis of literature and of data. All authors read and approved the final manuscript.

## Funding

Partial financial support was obtained from the University of Calabria (*ex quota* 60%), from Fondazione Istituto Neurologico Nazionale (IRCCS) “Casimiro Mondino” (Ricerca Corrente 2017, Ministry of Health, Rome), Pavia (Italy) and from MIUR (PRIN 2017, protocol 2017XKJTLW). DS received financial support from the European Commission in the frame of the FSE (Fondo Sociale Europeo) and from Calabria Region to complete her Ph.D. at the University of Calabria and the data concerned with experiments carried out with 2 and 6 month old mice have formed part of her Ph.D. thesis but have never been published before in any form or in any scientific Journal.

## Conflict of Interest

The authors declare that the research was conducted in the absence of any commercial or financial relationships that could be construed as a potential conflict of interest.
